# Caring Stress Management: A Qualitative Study of Strategies Used by Iranian Nurses

**DOI:** 10.1002/nop2.70778

**Published:** 2026-08-27

**Authors:** Amir Hossein Goudarzian, Elham Navab, Hamid Sharif‐Nia, Rebecca H. Lehto, Alireza Nikbakht Nasrabadi, Omolhoda Kaveh, Sara Alavi, Pouya Farzami

**Affiliations:** ^1^ Department of Psychiatric Nursing, Nasibeh School of Nursing and Midwifery Mazandaran University of Medical Sciences Sari Iran; ^2^ School of Nursing and Midwifery Tehran University of Medical Science Tehran Iran; ^3^ Department of Critical Care and Geriatric Nursing, School of Nursing & Midwifery Tehran University of Medical Sciences Tehran Iran; ^4^ Psychosomatic Research Center Mazandaran University of Medical Sciences Sari Iran; ^5^ Department of Nursing, Amol Faculty of Nursing and Midwifery Mazandaran University of Medical Sciences Sari Iran; ^6^ College of Nursing, Michigan State University East Lansing Michigan USA; ^7^ Department of Medical Surgical Nursing and Basic Sciences, School of Nursing and Midwifery Tehran University of Medical Sciences (TUMS) Tehran Iran; ^8^ Al‐Subtain University of Medical Sciences, International Branch of TUMS Karbala Iraq; ^9^ School of Nursing and Midwifery Mazandaran University of Medical Sciences Sari Iran; ^10^ Student Research Committee Mazandaran University of Medical Sciences Sari Iran

**Keywords:** caring stress, Iran, nurse, qualitative research, strategies

## Abstract

**Aim:**

To explore caring stress management among Iranian nurses.

**Design:**

A qualitative study using conventional content analysis explored Iranian nurses' experiences with caring stress management.

**Methods:**

Using purposive sampling, fifteen registered nurses with full‐time positions in high‐acuity clinical units across four public teaching hospitals in Tehran, Iran, participated in individualized semi‐structured interviews. Data were analysed using conventional qualitative content analysis following an established framework.

**Results:**

Managing caring stress involved a multi‐layered process. Nurses engaged in professional interactions. sought organizational support, focused on enhancing their clinical care knowledge and skills, and utilized rigorous time and care management. Strategies also included establishing effective therapeutic communication, dedicating more time to follow‐up care, practicing psychological care to build self‐confidence, and trying self‐care practices. The ability to manage caring stress was not uniform; with nurses describing significant structural and contextual barriers. Heavy workloads, severe nursing shortages, compressed shift schedules and lack of systematic institutional support frequently disrupted their capacity to consistently implement caring stress management, with some describing episodic severe emotional weariness and ethical dilemmas in isolation.

**Conclusion:**

The findings suggest that caring stress is a complex, multifaceted phenomenon influenced by a dynamic interaction among individual, professional and organizational factors. Effective caring stress management doesn't rest solely on individualized coping efforts; it is a larger system process that requires an institutional culture that prioritizes the importance of nursing well‐being alongside clinical duties. System‐level challenges, exacerbated by resource constraints, need addressing to bolster nursing resilience, minimize medical errors and sustain the Iranian nursing workforce.

**Clinical Implications for the Profession:**

Caring stress management is recommended to be integrated into institutional culture. Since nurses are vital stakeholders in patient safety, caring stress may directly or indirectly threaten care quality. A more supportive, resource‐balanced approach could help nurses cope with daily caring demands. Healthcare administrators may consider structured team‐building initiatives, peer support networks and simulation‐based training. Furthermore, nurses could benefit from being offered concrete self‐care provisions independently, such as flexible scheduling, dedicated relaxation spaces and accessible psychological counselling services.

**Reporting Method:**

The study adhered to EQUATOR guidelines and the COREQ checklist for qualitative studies.

**Patient or Public Contribution:**

No patient or public contribution.

## 1 Introduction

Professional nursing in Iran is a career choice that may adversely affect physical and mental health due to ongoing practice‐related stressors (Aloufi et al. 2021). Such stressors are imposed by direct ongoing contact with critically ill patients and distressed families and often overwhelming workloads. Chronic work‐related stress is recognized to affect nursing performance, job satisfaction and patient outcomes (McKinless 2020). Research has reported that over half of the nursing workforce reports high levels of work‐related stress and are susceptible to developing health conditions such as hypertension and depression. Given high levels of chronic stress contribute to medical errors and reduced patient satisfaction, evaluating caring stress is essential towards improving practice and patient care (Aslan and Pekince 2021).

Occupational stress emanates from workload demands and external environment pressures whereas caring stress arises from the emotional impact that is associated with empathizing and understanding the suffering of patients and their families (Babapour et al. 2022). Caring stress involves the strong interpersonal dynamics that are an essential component to providing nursing care. Caring stress is differentiated from mental or emotional stress that is a result of personal issues, as it is activated within the professional field secondary to ethical dilemmas and emotional weariness (Goudarzian et al. 2024). Caring stress places the nurse in a tenuous position of managing their deeply personal compassion for patients and families' wellbeing against their personalized capacities to promote it (Goudarzian et al. 2024).

Although stress‐related concepts are widely discussed in nursing literature, caring stress represents a distinct phenomenon that warrants conceptual clarification. Occupational stress generally refers to psychological and physiological strain arising from workplace demands, organizational pressures, workload, role ambiguity, or resource limitations. In contrast, caring stress originates specifically from the responsibilities and challenges associated with maintaining responsibility for patients' well‐being during care provision (Goudarzian et al. 2024).

Caring stress also differs from burnout, compassion fatigue and moral distress. Burnout is typically conceptualized as a long‐term outcome of chronic occupational stress characterized by emotional exhaustion, depersonalization and diminished professional accomplishment (Babapour et al. 2022). Compassion fatigue primarily reflects the emotional burden resulting from repeated exposure to patients' suffering and trauma, whereas moral distress arises when healthcare professionals are unable to act according to their ethical judgements because of institutional or contextual constraints (Garnett et al. 2023). While these phenomena may coexist with caring stress or emerge as potential consequences of prolonged caring stress, they do not fully capture the broader caring demands experienced by Iranian nurses (Goudarzian et al. 2024).

Drawing on the previously published conceptual synthesis, caring stress can be understood as a multidimensional form of stress that emerges directly from the process of caring for patients. It encompasses clinical responsibilities, emotional involvement, professional accountability, ethical obligations, interpersonal interactions and organizational challenges encountered during caring activities (Goudarzian et al. 2024). The concept is therefore best understood within the framework of transactional stress theory, which views stress as the result of dynamic interactions between individuals and their environments. Within this perspective, caring stress arises when nurses perceive caring demands as exceeding available personal, professional, or organizational resources. Consequently, caring stress reflects not only exposure to stressful conditions but also nurses' appraisal of caring responsibilities and their ability to manage them effectively (Babapour et al. 2022).

### 1.1 Context of Caring Stress in Iran

Iranian nurses face immense occupational stress due to systemic challenges, which have worsened secondary to economic sanctions. Limited access to essential medical supplies makes it difficult for nurses to provide proper care (Ahmadi Chenari et al. 2020). For example, shortages of personal protective equipment and critical care supplies compromise both patient and nurses' wellbeing, particularly during public health crises such as occurred with the COVID‐19 pandemic, that increase vulnerability to work‐related infectious exposures (Barasteh et al. 2021). Reductions in nurse‐to‐patient ratios compound Iranian's hospital workforce stress due to sustained pressures to meet untenable patient care demands that contribute to burnout, fatigue and diminished job satisfaction (Shamsi and Peyravi 2020). Not being able to provide proper care because of limited resources compounds overwhelm and perceptions of inadequacy, and increases the experience of moral distress due to the witnessing of preventable suffering and deaths (Ahmadi Chenari et al. 2020).

Caring stress management is a critical potentially modifiable element to avert the possible deleterious consequences associated with occupational stress and to promote the possibility of psychological health and career satisfaction (Khazar and Jalili 2020). When nurses adhere to psychological self‐care principles and practice stress management, a balanced scenario for personal well‐being is optimized (Khazar and Jalili 2020). Managing caring stress is crucial for nurses to be adjusted, satisfied with their job, and to experience overall well‐being. Utilizing effective management techniques and prioritizing psychological health may thus yield improvements in perceived mental well‐being and job satisfaction (Goudarzian et al. 2024).

Stress management involves strategies to reduce and control stress in the workplace. Previous research has highlighted work‐related stressors like shift changes and heavy workloads, but the caring stress that stems from direct patient care is recognized as a common source of stress for nurses worldwide (Betke et al. 2021; Ofei et al. 2020). This aspect, despite being of such importance, has not been duly studied by the scholars.

High levels of stress among nurses contribute to decreases in empathy and increases in occurrence of professional errors, which detracts from the quality of healthcare delivery and patient satisfaction with the care provided (McKinless 2020). Much literature has focused on stress‐reduction strategies as an intervention in the management of caring stress (Aloufi et al. 2021; Ofei et al. 2020). However, more attention is needed to shift the focus from occupational stress to caring stress itself and towards effective strategies for managing the same (Goudarzian et al. 2024). Thus, this qualitative study addresses this gap by delving into the concept of caring stress and its management strategies via an evaluation of what causes stress for Iranian nurses, how they experience and cope with it and their most common stressors and effective coping mechanisms. The study also seeks to provide recommendations for interventions to better support nurses in managing their stress (Goudarzian et al. 2024).

Accordingly, the study was guided by the following research questions:

1. What strategies do Iranian nurses use to manage caring stress?
2. What individual, professional and organizational factors influence the use of these strategies?

## 2 Materials and Methods

### 2.1 Design

A qualitative approach was used to explore Iranian nurses' experiences of caring stress management. Data were collected through semi‐structured interviews and analysed using conventional content analysis through identifying meaningful associations, without reliance on existing theories (Hsieh and Shannon 2005). The data analysis process involved repeated readings to ensure a thorough comprehension of the information gathered (Hsieh and Shannon 2005). This research method is utilized when existing theories or literature about the concept under study are limited, and this knowledge is either incomplete or requires further description (Erlingsson and Brysiewicz 2017). Although caring stress is a central and significant concept associated with occupational stress among nurses, limited attention and research have been focused on this conceptual area (Goudarzian et al. 2024). There is a need to reevaluate existing assumptions and explore new dimensions within the Iranian and Islamic cultural context to analyse the concept of caring stress, its formation among nurses, and its dimensions based on personal perspectives and experiences. Additionally, as a novel concept, the management of caring stress needs thorough examination. Therefore, to clarify and explain the concept of caring stress management, and to identify its related constructs, the method of conventional content analysis was employed.

### 2.2 Setting and Contextual Characteristics

This study was conducted across four major public teaching hospitals affiliated with Tehran university of Medical Sciences in Tehran, Iran, between September and December 2024. These institutions serve as primary tertiary referral centers in the metropolitan capital, capturing a highly diverse socio‐demographic patient population. Data collection was focused in high‐acuity clinical units including the Emergency Departments (ED), inpatient Intensive Care Units (ICU), Coronary Care Units (CCU) and Oncology wards where acute, unpredictable patient conditions significantly amplify daily caring challenges. The organizational environments of these public facilities are heavily characterized by systemic resource constraints and severe structural imbalances. Further, due to strict economic sanctions and national nursing shortages, these hospitals operate under a harsh nurse‐to‐patient ratio (frequently ranging from one nurse to four to six patients in intensive care and up to 12 patients in general acute care medical‐surgical wards during night shifts). Further, the nurse workload characteristics are distinctively demanding, featuring compressed shift schedules, high patient turnover rates, a high volume of complex/unstable admissions and systemic shortages of critical personal protective equipment (PPE) and advanced medical supplies. This demanding and dynamic baseline environment means that nurses routinely and personally navigate ongoing high cognitive load, challenging ethical dilemmas and cumulative emotional weariness alongside their active clinical duties.

### 2.3 Sampling

Fifteen nurses were recruited using purposive sampling with a maximum variation strategy to ensure representation of diverse nursing experiences and perspectives regarding caring stress management. Variation in sampling was sought in terms of age, gender, educational level, years of clinical experience, hospital wards and organizational positions. Thus, the sample included nurses who worked in diverse clinical environments and who possessed varying levels of professional background, enabling an exploration of a broad range of personalized experiences related to caring stress and coping strategies.

Inclusion criteria were: (1) being a registered nurse working full‐time in the selected hospitals, (2) having at least 1 year of clinical experience because, according to Benner's novice to expert theory, these nurses have moved beyond the novice stage and engage more independently, thus having more experience with the concept of caring stress management (Mortimore et al. 2021), and (3) willing to participate in the study. Exclusion criteria included self‐reported psychological disorders such as clinical depression and/or mood disorders, history of active substance use including alcohol consumption, and inability to participate in interviews.

Interviews lasted between 30 and 60 min, depending on the contextual situation and mutual agreement. The time and place of the 15 interviews were determined based on the nurses' schedule and preferences. All interviews were recorded and were conducted in private consultation rooms free of distractions and intrusions.

### 2.4 Data Saturation

Data collection and analysis were conducted concurrently. Following each interview, transcripts were reviewed, coded and compared with previously collected data to identify new codes, categories and conceptual insights. Saturation was assessed continuously through team discussions following each interview during the analytic process.

Preliminary saturation was achieved after the 13th interview, when no substantially new codes or categories emerged from the data and participants' accounts largely confirmed previously identified patterns. To ensure the completeness and stability of the findings, two additional interviews were conducted. These interviews yielded no new conceptual information and served to confirm completion of data saturation. Therefore, the final sample consisted of 15 participants.

The adequacy of the sample size was determined based on informational richness and saturation rather than a predetermined numerical target. Consistent with qualitative research methodology, recruitment was discontinued when additional interviews no longer contributed meaningful new insights to the emerging conceptual categories (Hennink and Kaiser 2022).

### 2.5 Data Collection Procedures

Following ethical approval from the human subjects committee, participants were selected based on the inclusion criteria and signed informed consent was obtained for the audio‐recordings and interviews. Following scheduling of the time and place of the interviews based on participant preferences, the purpose of the interview was reiterated, and participants were assured of the confidentiality of their information and the security of the recorded audio. Additionally, given their rights as volunteering participants, they were informed that interviews could be discontinued at any time and that responding to any of the questions was entirely by choice. At the end of each interview, participants were asked if they could be contacted in case follow‐up interviews or confirmation of their statements were needed.

After each interview, the recorded content was transcribed verbatim. The interviewer listened to the recordings multiple times and reviewed the written transcripts repeatedly to gain a deeper understanding of the participants' feelings towards their experiences. Observations were noted alongside the transcripts and analysed (Tenny et al. 2017). The semi‐structured interviews were conducted using a standardized interview guide, which included open‐ended questions focused on the study's objectives and key concepts related to caring stress management (Bengtsson 2016).

Examples of questions posed during the interviews included: ‘Can you talk about the care you provide for your patient?’ ‘What causes you to feel stressed when caring for your patient?’ ‘What are the signs and symptoms of stress you experience as a result of caring?’ ‘What actions do you take to alleviate stress while caring for your patients?’ and ‘How do you manage the stress arising from caring for your patient?’

### 2.6 Data Analysis

Data were analysed using the conventional content analysis approach proposed by Elo and Kyngäs (2008). Data collection and analysis were conducted concurrently to allow emerging insights to inform subsequent interviews. During the preparation phase, audio recordings were transcribed verbatim and transcripts were read and reviewed repeatedly to achieve immersion in the data and gain a holistic understanding of the content. Meaning units relevant to experiences of caring stress management were identified, consisting of words, sentences, or paragraphs containing related content that reflected a single idea or experience.

During the organization phase, the identified meaning units were condensed while preserving their core meaning and were assigned initial codes. Coding was conducted line‐by‐line whenever appropriate to ensure that important details and nuances were captured. Similar codes were then compared and grouped according to conceptual similarities and differences. Through an iterative process of constant comparison, related codes were organized into subcategories. Subcategories with shared meanings were subsequently clustered into broader categories representing higher levels of abstraction.

Finally, categories were reviewed and interpreted to identify overarching themes that reflected the latent meaning of participants' experiences. Theme development involved continuous movement between the original transcripts, codes, categories and emerging interpretations to ensure consistency and data fidelity. Throughout the analytic process, the evolving coding structure and thematic framework were systematically reviewed to confirm that the generated themes accurately represented participants' accounts.

Two researchers with a qualitative background independently reviewed all transcripts and generated preliminary codes before comparing interpretations. If disagreement occurred in coding, categorization, or theme interpretation, the original transcripts were revisited and interpretations were re‐evaluated until consensus was achieved. This iterative process enhanced the credibility, dependability and consistency of the findings.

MAXQDA version 10 software was used to organize interview transcripts, manage codes, facilitate retrieval of coded segments and support the development of categories and themes.

### 2.7 Credibility and Reliability

The trustworthiness of the study was established using the criteria proposed by Lincoln and Guba (1982), including credibility, dependability, confirmability and transferability.

Credibility was enhanced through prolonged engagement with the data, concurrent data collection and analysis and regular discussions among the research team throughout the analytic process. Participants were selected using purposive sampling with maximum variation to capture diverse perspectives and experiences related to caring stress management.

Dependability was supported through a systematic and transparent analytic process. All stages of data collection, coding, category development and theme generation were documented. Regular peer review of coding decisions and analytic interpretations was conducted throughout the study. Any differences in interpretation were discussed and resolved through consensus among the research team.

Confirmability was enhanced by maintaining an audit trail documenting methodological decisions, coding procedures, category development and analytic reflections. The research team engaged in reflexive discussions throughout the study to consider how researchers' backgrounds, assumptions, and experiences might influence data interpretation.

Transferability was strengthened through purposive maximum variation sampling and the provision of detailed descriptions of participants, study context, data collection procedures and analytic processes. Further, preliminary findings, coding structures and thematic interpretations were reviewed and discussed with qualitative research experts to challenge assumptions, consider alternative interpretations, and enhance analytic rigour. During data analysis, attention was given to both recurring patterns and also to divergent, contradictory and negative cases. These cases were examined alongside dominant themes to ensure that the findings reflected the full range of participants' experiences and to enhance the credibility and depth of interpretation.

### 2.8 Researcher Reflexivity

The research team consisted of nursing researchers with experience in both clinical nursing and qualitative research methods. Throughout data collection and analysis, researchers reflected on their professional experiences, personal assumptions and potential influences on their interpretations through regular discussions and analytic memo writing. Efforts were made to remain attentive to participants' perspectives and to ensure that interpretations emerged from the data rather than from pre‐existing personalized assumptions regarding caring stress and its management.

### 2.9 Ethics Approval

The study was approved by the ethics committee of Tehran university of medical sciences, Tehran, Iran (code: IR.TUMS.FNM.REC.1402.137). Written informed consent was obtained from all participants prior to the interviews, ensuring confidentiality and voluntary participation. All methods were performed following the guidelines and regulations of qualitative research. The transcripts were coded numerically without personal identifiers.

## 3 Results

Semi‐structured interviews were conducted with 15 nurses. From all the interviews, 7 categories and 16 sub‐categories were extracted, as shown in Table 1. Most of the participants possessed bachelor's degrees and ranged in age from 28 to 45 years old.

**TABLE 1:** Caring stress management concept among nurses.

Code	Primary categories	Subcategories
Receipt of help and support for caring management from more experienced colleagues	Professional cooperation and support	Professional interactions
Support from colleagues		
Provision of assistance from colleagues and physicians in care		
Engaging management and responsible authorities to support patient care decisions		
Improving interpersonalized professional interactions with colleagues (helping and getting help from colleagues)		
Asking for participation and help from colleagues		
Using colleagues' experiences to prioritize patient care measures	Sharing professional knowledge and experie nces	
Using experience to manage stress and guide others		
Modelling success in stress management from a colleague		
Talking and consulting with colleagues about patient care (getting guidance from colleagues)		
Referring care problems to superiors and receiving solutions from them	Professional guidance and support in stress management	
Reducing stress by asking for appropriate communication with colleagues and superiors		
Collaboration between nurses and failure to report minor deficiencies to expedite matters and advance patient care.	Team coordination and synergy for stress management	
Head nurse modelling of calmness and promoting calmness for other staff and provide more effective patient care		
Fair distribution of patients among nurses		
Reminding individuals of their assigned tasks for patient care		
Talking to colleagues to relieve stress and gain peace of mind	Emotional support and empathy	
Interacting with colleagues who create a sense of calm and avoiding people who cause a lot of stress		
Reducing stress through empathy and helping colleagues		
Enhancement of work proficiency	Gaining experience and caring skills	Enhancing knowledge and caring skills
Using past care errors as opportunities for personal growth and clinical learning.		
Adaptation through caring experiences		
Improving experience, communication skills and caring skills		
Staying up‐to‐date and increasing knowledge and skills as a life‐long learner.	Enhancing the level of caring knowledge	
Strengthening nursing knowledge to build trust		
Strengthening nursing knowledge		
Searching to strengthen care knowledge		
Reducing the stress of caring through gaining knowledge and staying up‐to‐date		
Reducing stress through the search for knowledge advancement		
Enhancing the care experience through increased knowledge and skills		
Time management in giving prescribed medications to patients	Prioritization in care	Time and care management
Prioritizing care interventions for patients		
Using colleagues' experiences to prioritize patient care measures		
Prioritizing caring activities		
Reducing caring stress by increasing focus and getting things done correctly	Mental management through self‐reflection	
Reducing stress through care management		
Prioritizing tasks and focusing on the most important patient care option		
Avoiding distracting situations and circumstances to increase focus and concentration on patient care		
Using a safe and quiet environment to write nursing reports to prevent distractions and increase concentration		
Focusing on care activities		
Maintaining calmness and patience with the patient and his/her companion especially if they are experiencing increased stress.	Constructive communication for maintaining calm	Effective therapeutic communication
Constructive interactions to promote calm and stress reduction for both the nurse and the patient and family.		
Establishing therapeutic communication and relationship with the patient		
Explanation with the patient to reduce the pressure and stress of care (effective communication with the patient's companions)		
Establishing appropriate communication with the patient and providing explanations and talking to the patient about treatment activities	Constructive communication for meeting patients' care needs	
Paying attention to the patient's wishes and interacting appropriately with the patient and his/her companion		
Monitoring the patient's medication intake to ensure adherence with medication instructions		
Increasing experience, communication skills and caring skills		
Better understanding of patients and being aware of the specific conditions of the patient and companions when interacting with patients with special conditions		
Brief but thorough information provision for patients and companions with provision of referrals to specialists if needed		
Assertiveness training: asking the patient's companions to leave the bedside if they are invoking caring stress.		
Spending time and energy on patients who are less compliant with treatment and medication to gain trust and improve communication.	Spending more time on patient care (devoting management)	Follow‐up care and commitment
Staying on subsequent shifts to complete assigned care tasks and follow up on patient matters with colleagues on the next shift		
Spending more time at work to complete caring tasks		
Being at the patient's bedside and following up on the patient's condition regardless of shift time	Continuous monitoring of patient condition	
Reducing stress through close and continuous monitoring of the patient		
Providing care with more care and peace of mind		
Reducing stress through increased self‐confidence following the use of trained facilities and skills	Increased self‐confidence	Psychological care to reduce stress
Increasing self‐confidence and reducing caring stress through spirituality		
Desensitization through caring experience		
Reduce stress by minimizing your skill deficits and addressing existing deficiencies and conditions.		
Reducing stress through self‐management (self‐control)		
Writing down thoughts about stressful occurrences and personal reflection on how to avoid them	Self‐reflection and self‐appreciation	
Using positive self‐talk		
Analysing circumstances such as unanticipated patient's death and avoidance of self‐blame.		
Self‐appreciation for effectively managing stressful situations (self‐appreciation)		
Focusing on the end of the shift to reduce caring stress		
Learning strategies to remain objective and to avoid indifference		
Rest during shift	Self‐care in various ways	Self‐Care
Infection control and personal protection during caring		
exercise engagement		
Mental health counselling		

As shown in Table 1, the use of caring stress management strategies varied depending on individual characteristics, work conditions and organizational context.

### 3.1 Professional Interactions

#### 3.1.1 Professional Cooperation and Support

Professional collaboration and support among colleagues was identified as one of the more effective strategies to reduce caring stress for the participants. Types of identified collaborations included decision‐making support, assistance with performing tasks and provision of support during challenging work situations. The experience and knowledge of more experienced colleagues can aid in managing these conditions more effectively. Participant 1 stated: ‘The most helpful thing seems to be having a skilled colleague who can provide support with the patient. This means when one is stressed, we can rely on that help’. *(ICU, 16 years experience)*.

Support from colleagues during busy work moments helps reduce stress and facilitates task completion. This support can include assistance with writing reports and/or sharing another nurse's workload. Participant 3 stated: ‘If you had a colleague who could cover for you, for instance by writing your nursing report when you needed to, it really helps to reduce one's stress in that moment’. *(CCU, 20 years experience)*.

Positive interactions with colleagues and supporting each other with daily work activities can have a significant impact on reducing stress. Participant 11 mentioned: ‘I try to get help from my colleagues in some tasks to reduce my stress, especially when I see a patient is in critical condition or if the work isn't getting done’. *(General unit, 2 years experience)*.

#### 3.1.2 Sharing Professional Knowledge and Experiences

The exchange of knowledge and experience among colleagues is another effective strategy for reducing stress. This process may involve consulting with coworkers, leveraging past experiences, or modelling the practices of successful nurses. Consulting and learning from others' experiences can help nurses prioritize their caring tasks, thus reducing confusion and enabling better stress management. Participant 2 noted: ‘Different colleagues use different approaches. For example, when I talk to a coworker and they share their experiences with me, it calms me down’. *(ENT, 6 years experience)*.

Observing and imitating the professional behaviours of successful colleagues is a strategy nurses use to improve their skills and reduce stress. Participant 6 explained: ‘During my time in clinical practice, I've always observed how successful individuals around me handled situations… Over time, I've started applying what I believe made them more successful to my own professional life’. *(Surgery, 15 years experience)*.

Guidance and support from management can help nurses make better decisions in critical situations and feel more secure. In complex and challenging circumstances, nurses often seek advice and support from their supervisors or managers. Participant 6 noted: ‘It's not like I'd bring up the issue with a junior colleague… I prefer to discuss it with individuals who are in higher positions than me’. *(Surgery, 15 years experience)*.

Leveraging the experience and knowledge of management can lead to more effective resolution of caring challenges. Participant 5 shared: ‘Personally, I involve my superior; I contact them and inform the doctor, saying something like, “Doctor, we have this many monitored beds, and your orders reflect this. Most of our patients need monitoring, so whoever is the higher priority should ideally get the monitoring”’. *(Emergency unit, 5 years experience)*.

#### 3.1.3 Team Coordination and Synergy for Stress Management

Coordination among team members and collective problem‐solving were identified as key factors for reducing stress and improving the quality of care. This coordination involves task delegation and working together to quickly resolve issues. Collaboration and harmony within the team can significantly alleviate stress during unanticipated emergency situations. Participant 12 remarked: ‘Mistakes can happen on the emergency side… we try to resolve the issue among ourselves’. *(PICU, 11 years experience)*.

Equitable distribution of assignments among nurses helps prevent unnecessary pressure. Ensuring a balanced workload allows nurses to focus more effectively on their responsibilities, reducing the likelihood of errors, especially in critical care situations. Participant 3 shared: ‘I need to consider that when a new patient is admitted, I shouldn't assign them to someone already overwhelmed. Instead, I might delegate or assign two people to handle the new patient, so I don't stress out the other nurse. Otherwise, their care for their critical patient might suffer’. *(CCU, 20 years experience)*.

#### 3.1.4 Emotional Support and Empathy

Creating an empathetic atmosphere and providing mutual emotional support from colleagues and managers can play a key role in reducing stress. Having an appropriate space to express emotions and engage in conversations with coworkers can help alleviate tensions. Participant 5 stated, ‘Talking it out and conversing with colleagues for emotional release helps gain peace of mind’. *(Emergency unit, 5 years experience)*.

Working with individuals who are more composed creates a better work environment and reduces stress. Participant 9 remarked, ‘Nurses and doctors who, at that moment… you know, give off a good vibe makes you want to always be around them. But there are others who really get on my nerves—let me put it bluntly, they're nitpickers—so I try to keep my distance from them’. *(Medical‐Surgical, 14 years experience)*.

Not all participants reported positive experiences. Some nurses described situations in which limited teamwork, communication difficulties, or lack of managerial support increased stress and reduced the ability to receive assistance. ‘Sometimes… for instance, everyone is so busy that there is no opportunity to ask for help. In those situations, you have to manage the stress on your own’. *(Participant 6, Surgery, 15 years experience)*.

### 3.2 Enhancing Knowledge and Caring Skills

#### 3.2.1 Gaining Experience and Caring Skills

Acquiring practical experience and skills was identified as one of the fundamental pillars in reducing stress and enhancing nurses' capabilities in care environments. Hands‐on experience, combined with repetition and learning from mistakes, was identified by nurses to gradually become more proficient in their work and to experience less stress. Having sufficient experience in the workplace enables nurses to perform their duties with greater confidence. Participant 1 mentioned: ‘Early in my service, when I started my internship, I was more stressed because I lacked sufficient experience. But during those moments when I wondered what to do, we were usually paired with an experienced person during shifts. This contributed to my expertise’. *(ICU, 16 years experience)*.

Learning from past mistakes can lead to reduced stress and improved performance. This approach helps nurses avoid repeating errors. Participant 4 noted: ‘Stress isn't good, but learning is. For example, if someone helped me understand that if I did a certain task differently, the issue could be prevented, I would make sure it never happened again’. *(ICU, 17 years experience)*.

With increased work experience, nurses can better respond to complex situations and utilize their skills effectively. Participant 8 shared: ‘In the beginning, when colleagues would say oncology patients are very pale and it's hard to find a vein, I felt stressed. But soon, I realized I could do it easily, and my confidence in finding veins improved’. *(Oncology, 2 years experience)*.

Practical experience not only strengthens clinical competencies but also enhances nurses' communication skills. These skills help reduce tension between nurses and patients. Participant 13 stated: ‘After working for two years, a person grows in every aspect—both in terms of communication skills, being able to talk with patients and their companions, and in terms of more efficient performance and better clinical skills’. *(Oncology, 2 years experience)*.

#### 3.2.2 Enhancing the Level of Caring Knowledge

Enhancing theoretical and skills‐based knowledge equips nurses to better handle complex and stressful situations. This improvement can be achieved through study, additional training courses, and utilizing scientific resources. continuing education is one effective tool for boosting nurses' confidence. Participant 4 stated: ‘I tried to improve my skills. For example, if my knowledge about CPR was limited and that caused me stress, I would study or ask experts’. *(ICU, 17 years experience)*.

Alignment with the goals of the medical team fosters trust between nurses and patients. Participant 5 remarked: ‘Otherwise, the patient won't trust you and might ask for another nurse, saying this one doesn't know anything. That's really bad. But when your answers align with the doctor's, the patient's trust increases’. *(Emergency unit, 5 years experience)*.

Using reliable resources and consulting with more experienced colleagues can reduce nurses' stress and help them perform better in critical situations. Participant 11 shared: ‘If I don't have the information, I ask. If there's something impactful, I consult with senior colleagues or the attending physician. Sometimes, during breaks, I browse reputable websites, which helps me feel calmer’. *(General unit, 2 years experience)*.

Efforts to learn and seek new information reflect nurses' commitment to their profession and to providing better care for patients. Participant 7 noted: ‘I try to show the patient that they matter to me. If, for instance, I don't know something, I'll find material or a book to explain it’. *(General unit, 13 years experience)*.

### 3.3 Time and Care Management

#### 3.3.1 Prioritization in Care

Learning how to prioritize is a key skill for nurses to adopt. This process involves organizing tasks based on their importance and focusing on the essential and urgent actions. For example, adherence to proper timing for medication administration can reduce stress but requires preparation time. Participant 2 noted: ‘This used to bother me… I would have to prepare the medication several times. Now I've learned to prepare them an hour in advance so I don't feel guilty about delays and experience unneeded stress’. *(ENT, 6 years experience)*.

Arranging care actions based on importance helps nurses manage their responsibilities more effectively. Participant 2 added: ‘Usually, when I have two or three patients at the same time, I try to handle the main tasks first. For example, I check their vital signs first, set up the IV, and then give the medications. This sequence reduces my stress’. *(ENT, 6 years experience)*. Collaboration and consultation with experienced colleagues can assist nurses in setting priorities. Participant 2 shared: ‘I talk to a colleague, and they share their experiences with me, like saying, “Prioritize” this first, and it'll be resolved’ *(ENT, 6 years experience)*.

Nurses prioritize addressing the most critical needs of patients based on their condition. Participant 3 explained: ‘When there's a high workload, bedside care is my top priority. I first complete the patient's bedside tasks to ease my mind, then move on to nursing reports and other tasks’. *(CCU, 20 years experience)*. Caring for patients in critical condition takes precedence. Participant 5 commented: ‘Among the patients we see, we prioritize those with critical conditions such as cardiac failure. This prioritization significantly reduces our worries’. *(Emergency unit, 5 years experience)*.

#### 3.3.2 Mental Management Through Self‐Reflection

Self‐Reflection is an effective tool for reducing nurses' stress during a hectic workday. This process involves focusing on essential tasks and analysis of past experiences. Concentrating on critical duties helped nurses work with greater precision and reduced stress. For example, Participant 10 noted: ‘During CPR, there's a lot of stress. In that moment, I'm very concentrated, I raise my voice, but I stay focused on the emergency’. *(Burn unit, 15 years experience)*.

Avoiding unnecessary interference was reported to also reduce stress for nurses. Participant 10 added: ‘When I manage my work well, I indirectly prevent others from interfering unnecessarily’. *(Burn unit, 15 years experience)*. Focusing on vital actions allows nurses to perform their duties with greater calmness and presence. Participant 14 shared: ‘I focus … and try not to get involved in other distractions’. *(Emergency unit, 3 years experience)*.

Further, avoiding workplace distractions also supports better concentration. Participant 4 explained: ‘To manage the stress of caring, I take myself to a place like the treatment room where no one can see me’. *(ICU, 17 years experience)*. Creating a quiet space for writing reports helps nurses maintain greater accuracy. Participant 2 mentioned: ‘I try not to sit so much at the station where I'm too visible. I go to the treatment room to write so I can focus more’. *(ENT, 6 years experience)*. Additionally, concentrating on primary caring duties and setting aside peripheral concerns reduces psychological pressure. Participant 11 stated: ‘I try to focus solely on the patient and their needs. That way, I feel more at ease’. *(General unit, 2 years experience)*.

### 3.4 Effective Therapeutic Communication

#### 3.4.1 Constructive Communication for Maintaining Calm

Establishing positive and effective communication with patients is a key strategy that was identified to both reduce perceived stress and enhance patient tranquillity. This communication includes soothing conversations and gaining patients' trust. Thus direct and sincere interactions are beneficial. Participant 10 stated: ‘Because I had background in mental health, I sit by the patient, talk, interact and manage the situation that way’. *(Burn unit, 15 years experience)*.

Fostering a sense of friendship was also acknowledged to reduce stress during patient interactions. Participant 7 mentioned: ‘Even if something happens, I always try to maintain a friendly approach with the patient. I believe that this creates a calm atmosphere, reducing both my stress and the patient's stress’. *(General unit, 13 years experience)*. A calm patient care approach with both the patient and their family members can also potentially reduce tension in the healthcare environment. Participant 8 said: ‘Most of the time, the patient's companion is under a lot of pressure, but if you remain calm and respond with kindness, we both will feel more at ease’. *(Oncology, 2 years experience)*.

#### 3.4.2 Constructive Communication for Meeting Patients' Care Needs

This type of communication focuses on addressing the patient's immediate needs and creating a sense of assurance. Nurses use this approach by providing clear and precise explanations about the treatment process. Participant 5 stated: ‘When I do an ECG, I approach with a friendly attitude. This lowers their tension’. *(Emergency unit, 5 years experience)*.

Responding to patients' and companions' questions builds trust and reduces stress. Participant 6 noted: ‘What I've seen during this time is that when you properly answer a patient's or their companion's question as a nurse, and guide them correctly, they calm down’. *(Surgery, 15 years experience)*. Accurate guidance and friendly behaviour ensure proper care delivery. Participant 7 shared: ‘Some patients with HIV don't easily accept treatment. You need to behave sensitively to convince them of the importance of their medication’. *(General unit, 13 years experience)*.

### 3.5 Follow‐Up Care and Commitment

#### 3.5.1 Spending More Time on Patient Care

Dedicating time and energy to caring for patients who are less compliant with treatment is essential for ensuring patient health. Some patients require more attention, especially when they resist medical treatment. Participant 1 remarked: ‘A main stress is about patients harming themselves or us, especially if they don't take their medications. We have to stay with them, talk and try to persuade them’. *(ICU, 16 years experience)*.

Some nurses prefer to extend their shifts to complete their care tasks and try to avoid stress incurred from unfinished duties. For example, Participant 13 reported: ‘I prefer staying longer to finish my work completely rather than leaving tasks incomplete, because it would stress me out’. *(Oncology, 2 years experience)*.

#### 3.5.2 Continuous Monitoring of Patient Condition

Careful and continuous monitoring of patients' conditions is a crucial commitment for nurses that not only reduces their stress but also improves care quality. Some nurses emphasize that they cannot leave their duties without ensuring the patient's condition is stable, even if their shift has ended. Participant 9 mentioned: ‘I was supposed to leave at 12:30 but stayed until 3:30 to make sure the patient's condition stabilized. That's the only way I can relax’. *(Medical‐Surgical, 14 years experience)*.

Occasionally, patient conditions demand extra attention, even if it means additional shifts. Participant 3 said: ‘I stayed at the hospital until 10 PM despite being on the night shift. It was exhausting, but monitoring the patient's condition reduced my stress’. *(CCU, 20 years experience)*. Regular check‐ups and ensuring patient safety lower nurses' stress. Participant 11 shared: ‘I try to frequently check on patients or contact someone listed in their records to ensure they are cared for. This reduces my stress’. *(General unit, 2 years experience)*.

### 3.6 Psychological Care to Reduce Stress

#### 3.6.1 Increased Self‐Confidence

Confidence is a key factor in reducing nurses' stress. It stems from experience, knowledge and preparedness for challenging care situations. Nurses can effectively manage work stress by being well‐prepared and leveraging their skills. Participant 10 remarked: ‘In the workplace, the most important thing is having confidence. It reduces stress. Before approaching a patient, I quickly review the necessary tools and tasks based on my experience and studies’. *(Burn unit, 15 years experience)*.

Some nurses find solace and increased confidence through their spiritual beliefs. Participant 9 shared: ‘I felt under immense stress, but I did my work correctly while calling upon God. The moment is still vivid in my memory’. *(Medical‐Surgical, 14 years experience)*. Experience in handling challenging situations helps nurses develop courage and experience lower perceived stress. For example, Participant 7 noted: ‘Once you've experienced a needle stick, the second time you become more confident and less stressed’. *(General unit, 13 years experience)*.

By managing emotions and behaviours, nurses can remain calm and make better decisions during stressful situations. Participant 12 mentioned: ‘When conditions become tough, I try to stay composed, which helps me make better decisions and reduces stress’. *(PICU, 11 years experience)*.

#### 3.6.2 Self‐Reflection and Self‐Appreciation

Self‐reflection and self‐appreciation were also identified as effective tools for reducing stress. Analysing experiences and celebrating successes help nurses learn and grow. Writing down stressful events and exploring improvement strategies were common practices among nurses. For example, Participant 13 shared: ‘After a stressful shift, I write about the day's events and analyze what I could have done differently’. *(Oncology, 2 years experience)*.

Positive self‐talk is another effective stress reduction method. Participant 9 said: ‘I used to reassure myself internally, saying ‘don't worry, it will be fine.’ Even with little experience, this approach worked’. *(Medical‐Surgical, 14 years experience)*. Rewarding oneself after managing a challenging situation motivates nurses and reduces stress. Participant 6 shared: ‘Once I'm sure the crisis is resolved, I treat myself to something nice as a reward’. *(Surgery, 15 years experience)*. Facing stressful conditions repeatedly helps nurses become more accustomed and react more calmly. Participant 12 noted: “When the environment is chaotic, I tell myself it will pass and I feel less stressed.” *(PICU, 11 years experience)*.

### 3.7 Self‐Care

While some participants described engaging in self‐care practices such as short breaks, exercise, or counselling, others reported limited time, heavy workload, or lack of institutional support as barriers that restricted their ability to practice self‐care during or after shifts.

One simple yet effective method that was identified was taking short breaks during shifts. These brief breaks help nurses recharge and continue their tasks with a refreshed mind. Even pausing for tea can help alleviate stress. Participant 6 mentioned: ‘A short break, like having tea, helps refresh and distance me from the stressful environment’. *(Surgery, 15 years experience)*.

Maintaining hygiene and using proper protective equipment is another effective stress reduction strategy. These measures not only prevent disease transmission but also enhance nurses' sense of security. Participant 11 stated: ‘I ensure I follow safety protocols like wearing masks and gloves’. *(General unit, 2 years experience)*.

Regular physical activity was distinguished as a method to calm the mind and relieve daily tension. The practice of yoga and simple breathing exercises at work enhances relaxation. Participant 15 shared: ‘Since starting yoga, I've felt much calmer. Even at work, I practice simple breathing exercises that greatly reduce my stress’. *(ICU, 7 years experience)*.

Some nurses preferred consulting specialists to better manage their stress levels at work. The approach provides new perspectives on handling challenges. And supports understanding their problems and finding effective solutions. Participant 15 noted: ‘When I feel overly stressed and unable to cope, I consult a counsellor. This helps me gain a better perspective on my issues’. *(ICU, 7 years experience)*.

Nevertheless, several participants reported difficulty implementing self‐care strategies consistently because of shift work, fatigue, family obligations and staffing shortages. ‘I know exercise and rest would help, but after consecutive shifts I often do not have enough energy or time’. *(Participant 10, Burn unit, 15 years experience)*.

### 3.8 Variations and Barriers in Managing Caring Stress

Although participants identified numerous strategies for managing caring stress, their experiences were not uniform. Nurses differed in the coping approaches they preferred and in their ability to implement such strategies in their work environments. While some participants emphasized the importance of seeking support from colleagues and supervisors, others reported limited access to professional support because of heavy workloads, staffing shortages, or unsupportive workplace relationships.

Several participants also noted that certain coping strategies were difficult to maintain consistently. For example, although self‐care was widely recognized as important, often there was insufficient time and/or energy to engage in activities such as exercise, recreation, or psychological self‐care secondary to work demands and family responsibilities.

In addition, a minority expressed scepticism regarding the effectiveness of some stress‐management approaches. These nurses described situations in which stress persisted despite using coping strategies, particularly during periods of severe workload pressure, critical patient situations, or organizational challenges.

## 4 Discussion

The findings indicate that the use of caring stress management strategies among Iranian nurses can be impacted by a combination of individual, professional and organizational factors. The findings provide background to create a better understanding of caring stress management among Iranian nurses. The identified caring management strategies span three interconnected levels: organizational‐professional resources, clinical care management strategies and personal self‐care resilience resources with important overlapping content.

At the organizational‐professional level, supportive professional relationships and ongoing development of clinical knowledge and skills provide nurses with resources that enhance competence, confidence, and decision‐making capacity. At the clinical level, strategies such as prioritization of care, therapeutic communication and continuous patient follow‐up enable nurses to manage immediate caring demands more effectively. At the personal level, psychological self‐regulation and self‐care practices help nurses maintain emotional balance and recover from the cumulative pressures of caring.

Importantly, these levels do not function independently. Professional support may bolster nurses' confidence and enhance clinical decision‐making, which may also facilitate effective patient communication and care management. Similarly, successful clinical performance enhances self‐confidence and psychological resilience, creating a reinforcing cycle that mitigates caring stress. The findings therefore suggest that caring stress management is best understood as a dynamic and multilevel process rather than a collection of isolated coping behaviours.

### 4.1 Professional Interactions

The importance of professional interactions identified in this study is consistent with findings from qualitative studies conducted in diverse healthcare settings, where collegial support, teamwork and effective communication have been recognized as important resources for reducing caring stress among nurses (Agarwal et al. 2020; Fernandes and Tewari 2012). Studies suggest that supportive professional relationships help in creating an environment where nurses feel appreciated; therefore, reducing burnout (Fernandes and Tewari 2012).

Similar findings have been reported in both regional and international studies emphasizing the protective role of social and professional support networks (Fernandes and Tewari 2012). However, the present study extends this evidence by demonstrating that professional interactions serve not only as emotional support mechanisms but also as practical resources that enhance clinical decision‐making and confidence in patient care. In the Iranian context, where staffing shortages and high workloads are common challenges, reliance on informal peer support appeared particularly important for managing caring stress (Goudarzian et al. 2024). A supportive work culture contributes to lowered perceived stress and higher added value in patient care. Subsequently, training activities that emphasize team‐building and communication‐modification programs may enhance caring stress management.

The establishment of interdisciplinary teams may also strengthen professional interactions through the facilitation of a culture of mutual respect and learning from each other (Agarwal et al. 2020). Studies have shown that nurses perceive less stress when they have support and understanding from other healthcare professionals (Bradley and Cartwright 2002; Shemtob et al. 2022). Activities that enhance communication among the health‐related disciplines may also improve idea sharing and the development of collective care strategies, building confidence among nursing staff (Bradley and Cartwright 2002).

### 4.2 Enhancing Knowledge and Caring Skills

The emphasis participants placed on knowledge acquisition and skill development aligns with previous research demonstrating that professional competence is associated with increased self‐efficacy and reduced occupational stress among nurses (Amini et al. 2017; Cleary et al. 2011). However, unlike studies that conceptualize education primarily as a means of improving clinical performance, participants in the present study described knowledge and competence as mechanisms for reducing uncertainty and strengthening psychological confidence when facing complex caring situations. This finding highlights relationships between professional competence and caring stress management and suggests that continuing education may have both clinical and psychological benefits.

Determining the most vital components for advancement of professional growth in nurses that support caring stress management is essential. Self‐efficacy can be enhanced when nurses are well prepared to face complex clinical scenarios (Cleary et al. 2011). Providing mentorship opportunities for novice nurses may alleviate caring stress during their transition into practice (MacLean et al. 2017). Hospitals need to provide structured continuing education for practicing professional nurses in areas such as new technologies and treatment developments. Simulation‐based training for resuscitation techniques or crisis management, for instance, has been associated with lower perceived stress during real emergencies (Happell et al. 2013). Simulated scenarios can be used to build emotional readiness, allowing nurses to feel prepared when they face real‐life critical events (Happell et al. 2013; Ignacio et al. 2016).

### 4.3 Time and Care Management

Effective time management was identified as a major form of decreasing caring stress corresponding with research advocating time management training to improve efficiency and reduce stress (Ravari et al. 2020).

Structured tools such as checklists and electronic task organizers are promising in supplementing nurses' time management efforts. Such tools may enhance clarity regarding clinical responsibilities while also decreasing the cognitive load associated with competing task management.

Care coordination plays an important role in effective caring stress management. A fair and equitable task distribution may offset any one nurse feeling over‐burdened and resentful because of workload (Ravari et al. 2020). Pre‐shift planning meetings allow teams to allocate roles to coordinate care delivery and avert unanticipated true changes (Alkhawaldeh et al. 2020).

### 4.4 Effective Therapeutic Communication

Building effective therapeutic communication skills to ensure that interactions between the nurse and their patients and family convey empathy and support yield benefits by enhancing trust and respect (Amoah et al. 2019). Previous studies have identified that patient‐centered communication reduces perceived stress for both the provider and patient while also having a positive impact on care outcomes (Appiah et al. 2023; Vinitha 2022).

An important aspect of therapeutic communication is to adapt messaging style to the needs and emotional state of the patients. Active attentive listening improves understanding of patients' underlying concerns, thus fostering response patterns that can reduce anxieties (Amoah et al. 2019) and improve patients' treatment adherence (Sáez‐Ruiz et al. 2024).

Therapeutic communication was noted as protective against emotional exhaustion. By building purposeful and deliberate alliances with patients and their families, it may work to promote balance between professionalism and empathy in challenging situations that aggravate caring stress (Jelly and Rochmawati 2022).

### 4.5 Follow‐Up Care and Commitment

Personal investment highlighted the nurses' commitment to their professional work. Spending extra time with patients when available, even outside assigned responsibilities, indicated a high level of professionalism and commitment (Talaee et al. 2022). Intrinsic motivation to improve patients' health may also increase derived satisfaction with one's career and offset caring stress (Asgarnezhad Nouri and Soltani 2017).

Deep commitment is an expression of the moral and ethical values that the nurses upheld. The more nurses perceive their endeavoured actions as delivering a positive impact, the stronger their identity as a nurse becomes. However, while resilience is developed as an inner resource, nurses are vulnerable to emotional and cognitive depletion and tiredness (Asgarnezhad Nouri and Soltani 2017; Kamau et al. 2015).

### 4.6 Psychological Care to Reduce Stress

Psychological strategies such as reflective journalizing and mindfulness training may be important behaviours for managing caring stress. These findings are in line with evidence suggesting that mindfulness and self‐regulation techniques can be effective in reducing emotional distress (Gawande et al. 2023; Heeter et al. 2021; Scafuto et al. 2024).

Work environment design that supports self‐care approaches such as mindfulness‐based stress reduction may yield significant improvement to nurses' capacity to manage their psycho‐physiological stress responses (Ghawadra et al. 2019). Brief short mindfulness practices support awareness through emotional regulation and building of interoceptive awareness, and help nurses maintain focus even in highly stressful situations (Wexler and Schellinger 2023). Occupational access to mindfulness training through one's healthcare institution can create a culture where mental well‐being is prioritized. Training apps have been developed offering short cybermeditation programs that nurses may use to combat the caring stress that is endured in the work environment (Heeter et al. 2021).

### 4.7 Self‐Care

Finding organizational financial resources to support mental well‐being was emphasized as self‐care and as an efficient mechanism of coping (Stimpfel et al. 2022). Such practices have been shown to alleviate the physical and psychological strain that would, in turn, enable nurses to maintain their performance standards (Kravits et al. 2010; Williams et al. 2022).

However, by tailoring appropriate self‐care practices, these behaviours could be improved even further. Abbreviated yoga and other breathing exercises incorporated into daily physical activities have contributed to reduced stress and generalized wellness (Kravits et al. 2010). Evidence suggests that short, consistent sessions of outdoor activity and also nature breaks may brighten the mood, boost energy and promote resilience in a high‐stress work environment (Kaleta et al. 2025; Williams et al. 2022).

Moreover, it is vital to promote the establishment of an organizational culture conducive to self‐care. This may include the provision of onsite wellness facilities, such as relaxation rooms and even fitness areas, which could encourage nurses to take brief, rejuvenating breaks during shifts. Research indicates that such initiatives may not only reduce burnout but also increase job satisfaction and retention within nursing staff (Lloyd and Campion 2017).

### 4.8 Barriers and Contradictory Experiences in Managing Caring Stress

Although participants identified numerous strategies for managing caring stress, the findings also suggest that the effectiveness and accessibility of these strategies were not uniform across situations and settings. Several participants described circumstances in which commonly reported coping approaches were difficult to implement because of often intense heavy workloads, staffing shortages, time constraints and the unpredictable nature of clinical care. For example, opportunities for professional consultation, reflective practice, or emotional support were limited during periods of high patient acuity or workforce shortages.

Furthermore, while supportive professional relationships were frequently described as valuable resources, some participants also reported interpersonal tensions, inadequate support from supervisors, or limited collaboration among healthcare team members. These experiences indicate that professional interactions may function as either protective or stress‐inducing factors depending on the organizational environment.

Similarly, strategies such as self‐care, stress reduction and psychological self‐management were not always easily achievable. Participants often acknowledged prioritizing patient care responsibilities over their own well‐being, resulting in limited time for rest, exercise, counselling, or other self‐care activities. In some cases, nurses described continuing to work despite significant fatigue or emotional burden because of professional obligations and staffing constraints.

These contradictory experiences suggest that caring stress management should not be viewed solely as an individual responsibility. The effectiveness of coping strategies depends substantially on organizational climate, availability of resources, supportive leadership and workplace culture. Recognizing these barriers provides a more balanced understanding of caring stress management and highlights the need for system‐level interventions alongside individual coping efforts.

### 4.9 Clinical Implications

The study results provide significant implications for nursing practice, health care systems and health policy‐making in Iran. Understanding the specific aspects of the management of caring stress among Iranian nurses helps to specify domains that may benefit from enhancements at the different levels of human and organization. Organizational support and team collaboration were important factors influencing the caring stress. Health care institutions may consider adopting structured support such as interdisciplinary team building, peer support networks, and supervisory engagement to develop a supportive context. Skills preparation will require an arrangement for regular simulation training, mentorship programs and continuing education such as the use of simulation to prepare nurses for high‐stress scenarios could increase confidence and proficiency, particularly for less experienced nurses. By implementation of effective time management strategies such as structured workflows and fair workload distribution may reduce the mental burdens of their duties.

Therapeutic communication and psychological resilience are both helpful towards managing caring stress. Patient‐centered care initiatives may enhance communication skills, build trust and reduce anxiety among both nurses and patients. Mindfulness‐based techniques, complemented by self‐reflective practice, may facilitate emotional regulation and concentration among nurses in stressful circumstances. Addressing issues of self‐care practices, like wellness initiatives, relaxation areas and flexible scheduling, may strengthen nurses' wellbeing – physically and mentally (Lehto et al. 2020).

Public outcry for policy advocacy requires investment in healthcare infrastructure to address systemic challenges, such as resource shortages and economic constraints. These measures improve the well‐being of nurses while enhancing the quality of care provided to patients by minimizing medical errors, increasing satisfaction and ultimately retaining skilled nursing staff. Multi‐faceted approaches are required in addressing caring stress among nurses for the sustainability of the health care workforce and better patient outcomes.

## 5 Conclusion

This research underlines the need for comprehensive approaches to managing caring stress in nurses by establishing the importance of organizational backing, upskilling, time management and psychological resilience. It suggests that the two features—distribution of nurse well‐being and patient care quality performance—give renewed advocacy to systemic reforms and targeted intervention against stressors.

By emphasizing the mental and physical well‐being of nurses regarding their contextual and cultural criteria, improved outcomes, such as job satisfaction, fewer errors and superior patient experiences could be leveraged to foster a healthier and more sustainable workforce for nursing.

This study contributes to the emerging conceptualization of caring stress as a distinct caring‐centered phenomenon that differs from broader occupational stress constructs. The findings suggest that caring stress management is a dynamic, multilevel ongoing process influenced by organizational, professional and personal factors. Future research should evaluate the proposed conceptualization in different healthcare settings and examine the cross‐cultural applicability of the findings in diverse nursing populations.

## Author Contributions

**Alireza Nikbakht Nasrabadi:** conceptualization, investigation, methodology, project administration, writing – original draft, writing – review and editing, supervision. **Elham Navab:** conceptualization, investigation, methodology, validation, formal analysis, writing – original draft, writing – review and editing. **Rebecca H. Lehto:** writing – original draft, writing – review and editing. **Pouya Farzami:** writing – review and editing, writing – original draft. **Hamid Sharif‐Nia:** conceptualization, writing – original draft, writing – review and editing, formal analysis, methodology. **Omolhoda Kaveh:** conceptualization, data curation, validation, writing – original draft, writing – review and editing, methodology, project administration. **Amir Hossein Goudarzian:** conceptualization, writing – original draft, writing – review and editing, data curation, validation, investigation. **Sara Alavi:** writing – original draft, writing – review and editing.

## Funding

The authors have nothing to report.

## Consent

Written informed consent was obtained from the nurses.

## Conflicts of Interest

The authors declare no conflicts of interest.

## Data Availability

The data that support the findings of this study are available on request from the corresponding author. The data are not publicly available due to privacy or ethical restrictions.
